# Incidentally cured psoriasis in a patient with refractory/relapsed diffuse large B-cell lymphoma receiving CD19 CAR-T cell therapy: a case report

**DOI:** 10.3389/fimmu.2024.1418768

**Published:** 2024-10-24

**Authors:** Song-yun Wang, Wan-hua An, Ze-song Wang, Wan-li Wang, Bin Zhang, Kai-lin Xu, Shu-li Guo, Ming Gao, Bo Li, Lei Huang, Huan-huan Tian, Wen-yi Guo, Hui-rui Wang

**Affiliations:** ^1^ Department of Hematology, The Second Affiliated Hospital of Henan University of Science and Technology, Luoyang, China; ^2^ Department of Hematology, Luoyang Central Hospital Affiliated to Zhengzhou University, Luoyang, China; ^3^ Central Laboratory, The Second Affiliated Hospital of Henan University of Science and Technology, Luoyang, China; ^4^ Department of Dermatology, The Second Affiliated Hospital of Henan University of Science and Technology, Luoyang, China; ^5^ Blood Diseases Institute, Xuzhou Medical University, Xuzhou, China; ^6^ Department of Dermatology, Luoyang Central Hospital Affiliated to Zhengzhou University, Luoyang, China

**Keywords:** case report, psoriasis, CD19 CAR-T, refractory/relapsed diffuse large B cell lymphoma, cell therapy

## Abstract

Chimeric antigen receptor T (CAR-T) cell therapy is a new treatment for cancers, but reports on curing immune-related skin diseases are limited. We report a case of successful CAR-T-cell therapy in a patient with refractory/relapsed diffuse large B-cell lymphoma (R/R DLBCL) who was incidentally cured of chronic generalized plaque psoriasis. The patient, a 65-year-old male who had a known history of psoriasis for 45 years, did not receive immunotherapy for psoriasis during this period. Imaging, molecular biology and immunology diagnostics confirmed DLBCL. After several weeks of standard-dose R-CHOP chemotherapy, the patient achieved partial remission, but according to CT, the patient relapsed, and there was no significant improvement in her psoriasis symptoms. Subsequently, the patient was enrolled in the CD19 CAR-T-cell therapy group. Four weeks after CAR-T-cell infusion, the patient’s abdominal pain disappeared, and there was a significant improvement in overall skin lesions. One year later, follow-up results indicated complete remission of R/R DLBCL (confirmed by PET-CT), with only minimal residual psoriatic skin lesions limited to the patient’s neck. The results of using CAR-T-cell therapy to achieve an incidental cure for psoriasis highlight the potential for exploring cell-based therapies for complex autoinflammatory skin diseases.

## Introduction

DLBCL is one of the most common and aggressive non-Hodgkin lymphomas (NHLs). In recent years, the introduction of R-CHOP (rituximab, cyclophosphamide, adriamycin, vincristine, and prednisone) chemotherapy has significantly improved the cure rate (approximately 50% to 60%) (1, 2). But approximate 30% to 40% patients will relapsed/refractory after received the first -line chemotherapy(R-CHOP), CD19 CAR-T will be the choice for second-line treatment, which has been reported that 52% to 93% patients have good response, and also the survival rate at one year later will get 48% to 83% (3, 4). The use of CAR-T cells for the treatment of immune-mediated systemic diseases has been reported in some cases. Recently, CAR-T cell therapy for SLE, has also been demonstrated to potentially modulate the immune response of the body, and the current scope of experimental treatments and follow-up in autoinflammatory diseases also indicates significant potential for widespread application (5, 6). In this case, we report for the first time a patient with R/R DLBCL who has a long history of psoriasis without receiving any systemic immunosuppressive treatment. Remarkably, after undergoing CD19 CAR-T cell therapy, the patient achieved control of DLBCL and experienced remission of chronic psoriasis. This finding contributes to our understanding of the relationship between lymphoma and psoriasis and suggests the potential for cell-based therapy in the treatment of psoriasis.

## Case presentation

**Figure 1:**
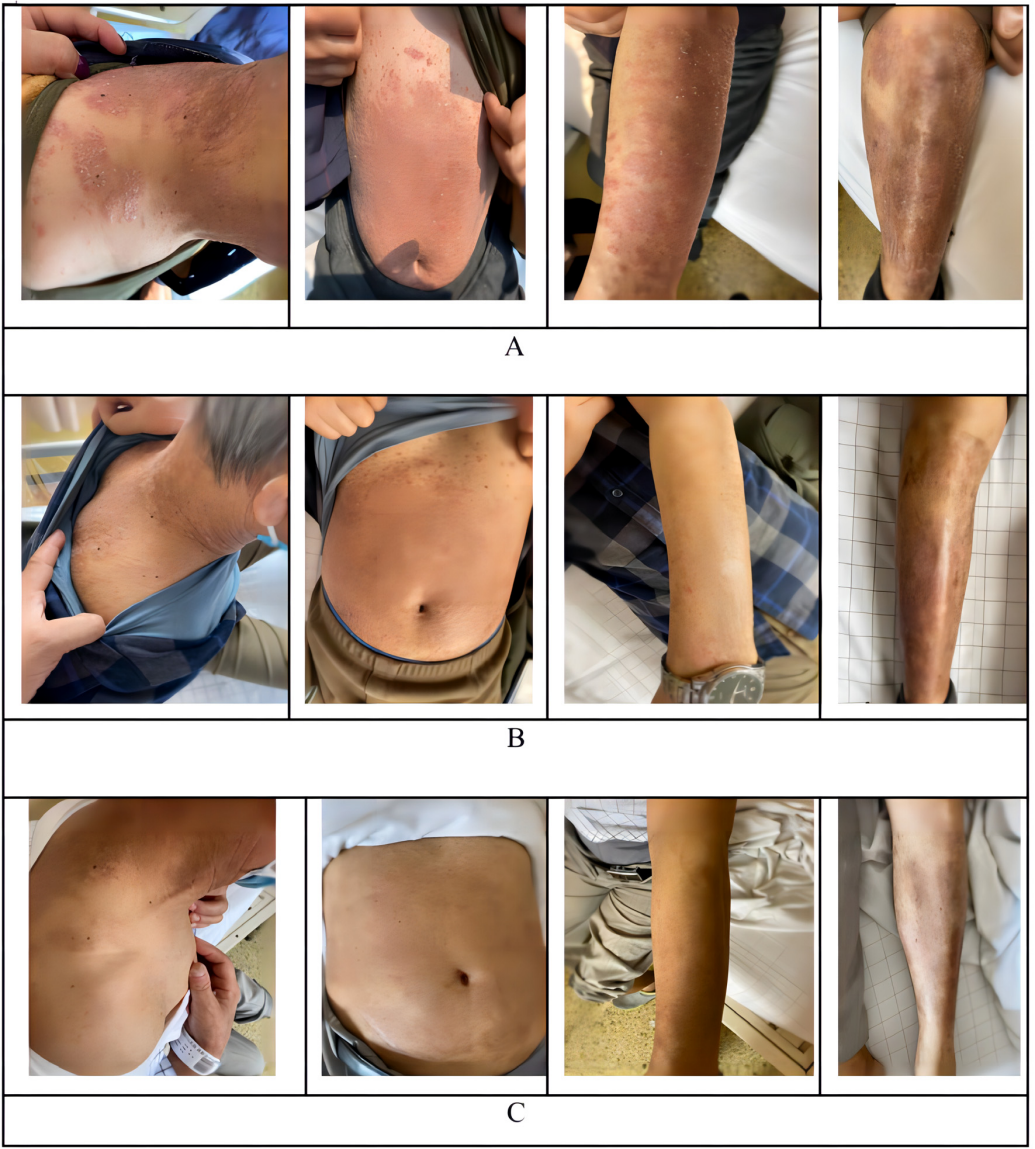
Observated of skin changes before and after infusion of CAR-T cells. **(A)** Skin changes before infusion of CAR-T cells. **(B)** +28 days Skin changes after infusion of CAR-T cells. **(C)** One year Skin changes after infusion of CAR-T cells.

A 65-year-old male with a known history of psoriasis for 45 years was initially admitted because of fever, fatigue, night sweats, and abdominal pain. Skin examination revealed diffuse flaky erythema of different sizes on the trunk and limbs with fused plaques associated with dry silvery scales, consistent with a known history of chronic generalized plaque psoriasis and a Psoriasis Area and Severity Index (PASI) of 64.8 (**Figure 1A**) and the Dermatology Life Quality Index (DLQI) (7–9) of 20,who was not particularly undergoing by any systemic therapies, including biologics or other immunomodulators. The patient’s affected areas by psoriasis covered the whole body and himself declare that no family history. However, it was felt that the systemic symptoms were unrelated to the psoriasis, and further clinical and pathological evaluation was pursued. CT imaging revealed an evidently thickened ileocecal wall, which prompted enteroscopic biopsy. Biopsy revealed ileocecal diffuse large B-cell lymphoma of the nongerminal center type. Immunophenotypically, the neoplastic cells were characterized as Bcl-2^+^ (80% +), Bcl-6^-^, CD10^-^, CD20^-^, CD3^-^, CD30^-^, CD5^-^, CD7^-^, c-Myc 30%, Ki-67 80%, Mum-1^+^, Pax-5^+^, and EBV^-^; *fluorescence in situ hybridization* (FISH) was positive for the Bcl-6 gene and negative for the C-myc and Bcl-2 genes. The bone marrow was not involved. Her lactate dehydrogenase (LDH) level was 302 IU/L, and the patient had a 46, XY karyotype. PET-CT revealed a five-point scoring system (5-ps) (10) score of 5 points and confirmed a thickened ileocecal wall (cumulative length 120.3 mm). These results combined with the patient’s medical history confirmed the occurrence of diffuse large B-cell lymphoma. No systemic lymphadenopathy was noted at the time of diagnosis.

**Figure 2:**
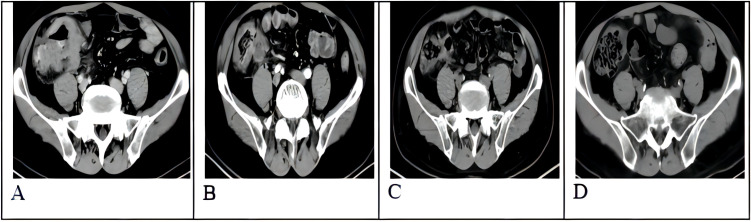
CT scan of tumor mass changes before and after infusion of CAR-T cells. **(A)** CT scan before infusion of CAR-T cells. **(B)** +28 days CT scan after infusion of CAR-T cells. **(C)** +75 days CT scan after infusion of CAR-T cells. **(D)** One year CT scan after infusion of CAR-T cells.

Treatment for his DLBCL was initiated with a first-line chemotherapy regimen of standard-dose R-CHOP for four cycles (which including 600mg rituximab), followed by partial response and relapse. Two cycles of rituximab were added for patients who achieved a poor response and progressive disease, including multiple enlarged lymph nodes on CT imaging in addition to a thickened ileocecal valve (**Figure 2A**). Notably, the patient’s affected areas of psoriasis did not show any notable changes (**Figure 1A**), except for the patient who reported mildly improved pruritus.

**Figure 3:**
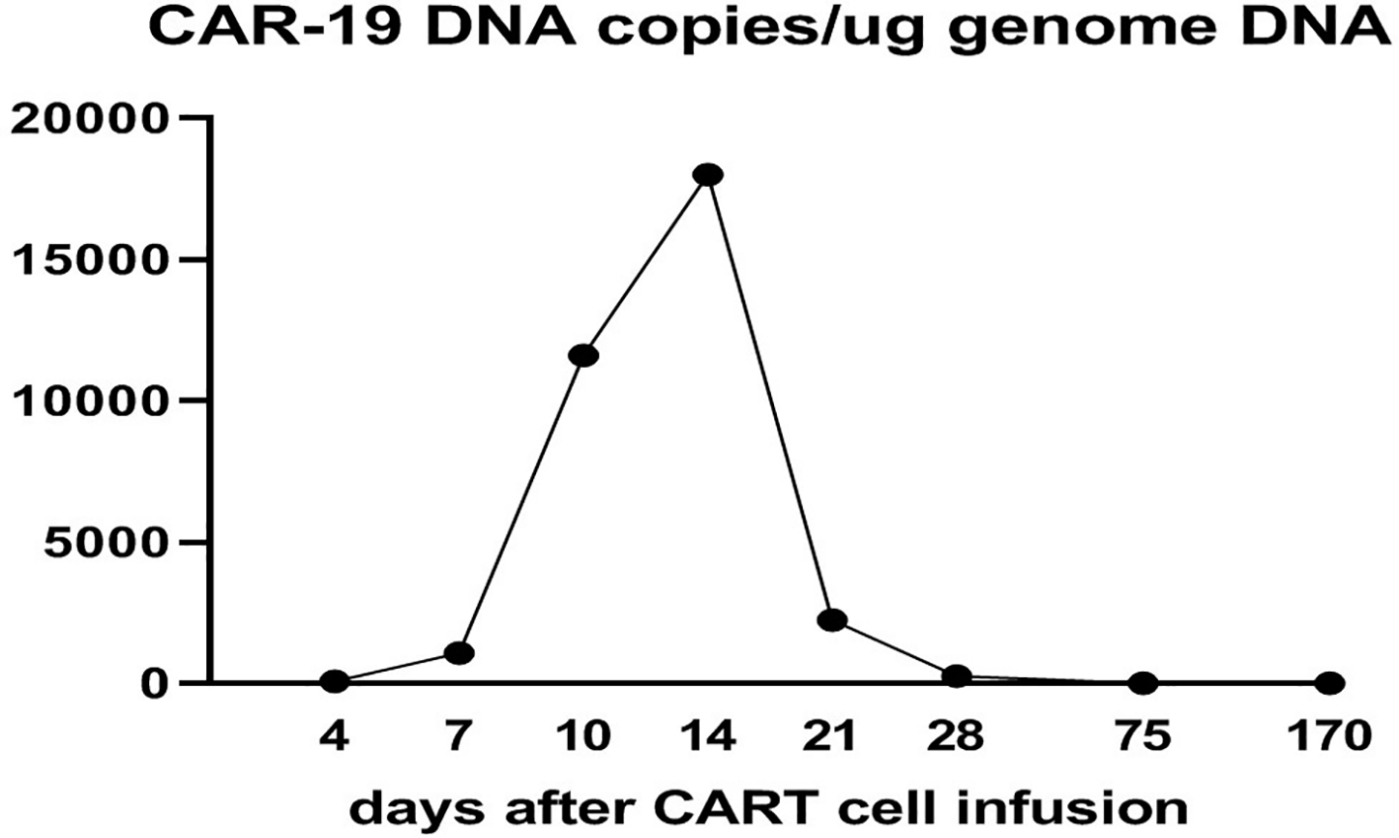
Expansion and persistence of CAR-T cells in peripheral blood after CD19 CAR-T infusion.

CD19 CAR-T cell therapy was initiated to treat the patient’s refractory DLBCL. The patient was infused with 2 × 10^6^/kg autologous CD19 CAR-T cells (Hebei Senlang Biotechnology Co., Ltd.; Clinical Trial Registration No.: CT04666168). Post-infusion grade 1 CRS occurred on days 7, 8 and 9 (+7 d, +8 d, and +9 d), after which the patient was treated symptomatically. The number of CAR-T cells was greatest at 14 d post-infusion, as shown in **Figure 3**. Four weeks after CAR-T cell infusion (+28 d), the patient’s abdominal pain disappeared (**Figure 2B**). Interestingly, during the same time period, the skin lesions significantly diminished, and post-inflammatory hyperpigmentation remained. The thick plaques gradually resolved with only rare fine scales on limited areas, and his pruritus significantly improved. These findings are consistent with the regression period of chronic generalized plaque psoriasis (**Figure 1B**), with a PASI score of 18.7.

With ongoing follow-up, the DLBCL was basically under controlled after 75 days of CAR-T infusion (**Figure 2C**). And one year after CAR-T-cell therapy, the treatment of DLBCL (complete remission based on PET-CT scan) was sustained (**Figure 2D**), and the patient had nearly completely resolved psoriasis with only mild post-inflammatory pigmentation and rare residual papules on his neck (**Figure 1C**), the PASI score was 4.8, and the decline rate: 92.59%, which also the DLQI score decrease to 2 and recent follow-up of 3.5 years the psoriasis showed no signs of recurrence (**Supplementary Figure 1**).

## Discussion

CAR-T as a cutting-edge immunotherapy, which has widely-used in hematologic cancer. With development of recent years, CAR-T has shown the surprising result in cardiac injury (Targeting FAP), myasthenia gravis (Targeting BCMA), SLE (Targeting CD19) and etc. (5, 11–14).

Psoriasis as a chronic recurrent immune-mediated inflammatory skin disease, but its definitive etiology and pathogenesis remain to be elucidated. It is usually considered as the cascade reaction of DC-Th17 in susceptible patients after receiving related risk factor to secrete large number of cytokines including IL17, IL23, TNF-alpha and INF-gama to induce activation of the keratinocytes, which drives the epidermal hyperplasia and the production of chemokines such as antimicrobial proteins and growth factor (15). These factors promote the characteristic changes of psoriasis, including angiogenesis, neutrophil infiltration and an increase in the number of helper T cell type 1 and Th17, creating a self-sustaining inflammatory cycle (16, 17). Through the study of the pathological tissue of the skin lesion of psoriasis, it is known that there are a large number of CD8 memory T cells residing in the skin lesion (18, 19). This feature also leads to the fact that after the end of the conventional systemic treatment, the memory T cells remaining in the skin lesion, upon receiving relevant stimulation, can directly reactivate the immune system in the tissue region, causing disease recurrence without recruitment of immune cells in blood (20). Current research evidence shows that psoriasis has a great correlation with the composition of the immune system in the microenvironment of the skin lesions and the cellular immunity dominated by memory T cells.

Study on dermatology biology in psoriasis have shown that the Dendritic Cell (DC) cooperate with pDC, Th1, Th1, macrophages, etc. to stimulate the keratinocyte activation and excessive proliferation through the IL23-IL17-TNF-alpha/INF-gama axis, thus producing the appearance of epidermal thickening (21–23). In this process, the differences in the abundance of different type of cytokines also produce different subtypes of psoriasis (17). Based on current knowledge, the biologic for psoriasis are mainly antagonistic to DC-Th17 axis related cytokines (24), such as Secukinumab (Anti-IL17), Ustekinumab (Anti-Human IL12/IL23), Adalimumab (Anti-Human TNF-alpha), and etc.

In recent years, with the in-depth research on psoriasis, the researcher has found that the interaction between T and B cells affects the process of immune diseases. A single cell and BCR sequencing data of PBMC showed that IgA1 or IgG1 plasma cells were significantly increased in psoriasis patients; moreover, the proportion of follicular T helper cell (Tfh) subset with high expression of Bcl-6 was relatively high in patients (25). Interestingly, the levels of circulating Tfh^CD3+CD4+CXCR5+^(cTfh) subsets ICOS+ or PD-1+, naïve B^CD19+IgD-CD27-^, activated B^CD19+CD86+^ cell and IL21 in peripheral blood were significantly positively correlated with PASI in psoriasis patient. More notably, activated B were significantly positively with cTfh and IL21 (26). These results suggest that B, cTfh and IL21 in peripheral blood may serve as important indicator of psoriasis progression.

CD19 and CD20 as the surface markers of B cells. Rituximab (Anti-Human CD20 type I) can bind to the B cell and causes B cell depletion. Some data shows that the incomplete B cell depletion often occurred in while using the rituximab during the treatment of autoimmune diseases (27–29), which push the scientist developed the CD19 CAR-T for lupus (13), has been achieved the remarkable clinical result (5, 14). In this case, the patient did not get any biologics or immunomodulators treatment before, but during the treatment of R/R DLBCL period, rituximab (dose: 600mg) was used for six cycle (4 cycles of R-CHOP, 2 cycles of rituximab alone), the data shows that the patients’ affected areas of psoriasis was not remission (**Figure 1A**), which suggested CD20 may was not a good target for psoriasis. In addition, the usage of rituximab also has a risk of inducing previous psoriasis (30).

Besides that, the patient was initially admitted to hospital for DLBCL. Since our medical team mainly treats DLBCL, we did not do too much pathological testing for the patient’s psoriasis. The microenvironment indicator such as cTfh, B cell or IL21 in skin lesion was lack of, which caused us failed to effectively proved the depletion of B cells was an effective method to cure psoriasis. But remission of psoriasis after CD19 CAR-T infusion showed that CD19 CAR-T cells are not only effective against B-lymphocyte tumors but also may change the abnormal immune state of patients with psoriasis. The infused CAR-T cells improved the skin lesions of the patient, which suggests that CD19-positive B lymphocytes may be involved in the occurrence and development of psoriasis. A previous study revealed a large amount of B lymphocyte infiltration in the affected skin of patients with psoriasis (31). There was a positive correlation between the PASI score and the number of CD19+ B cells in patients with psoriasis vulgaris and arthropathy (32). In particular, autoreactive skin-associated B cells (33) may reside in the skin and contribute to local chronic inflammation involved in the pathogenesis of psoriasis. These theories explain our case scenario: effective CD19 CAR-T-cell therapy depletes B cells, including autoreactive skin-associated B cells, leading to the resolution of severe psoriasis.

In conclusion, we reported for the first time the cure of psoriasis mediated by CD19 CAR-T, demonstrating that B cell depletion may play a role of cure psoriasis. Because of the surprising effect of CAR-T-cell therapy on severe psoriasis in addition to lymphoma, this case shed light on a novel and new therapeutic option for refractory psoriasis, which has always been challenging for dermatologists and may provide inspiring evidence for a better understanding of the immunological mechanism of psoriasis. The significance of these findings is limited by the nature of single case reports and the lack of further mechanistic exploration, which is warranted for future larger-scale and more in-depth studies. In addition, due to the CRS caused by CAR-T, the treatment plan of psoriasis may need to subtle designed and adjusted in the future, so as to benefit patients more safety.

## Data Availability

If necessary, the original data supporting the conclusions of this article will be provided by the authors.
