# Supplementary figures and images for: Incidentally cured psoriasis in a patient with refractory/relapsed diffuse large B-cell lymphoma receiving CD19 CAR-T cell therapy: a case report
Source: Front Immunol. 2024 Oct 24;15:1418768. doi: 10.3389/fimmu.2024.1418768 (PMC11555394; doi:10.3389/fimmu.2024.1418768)

## Slide 1
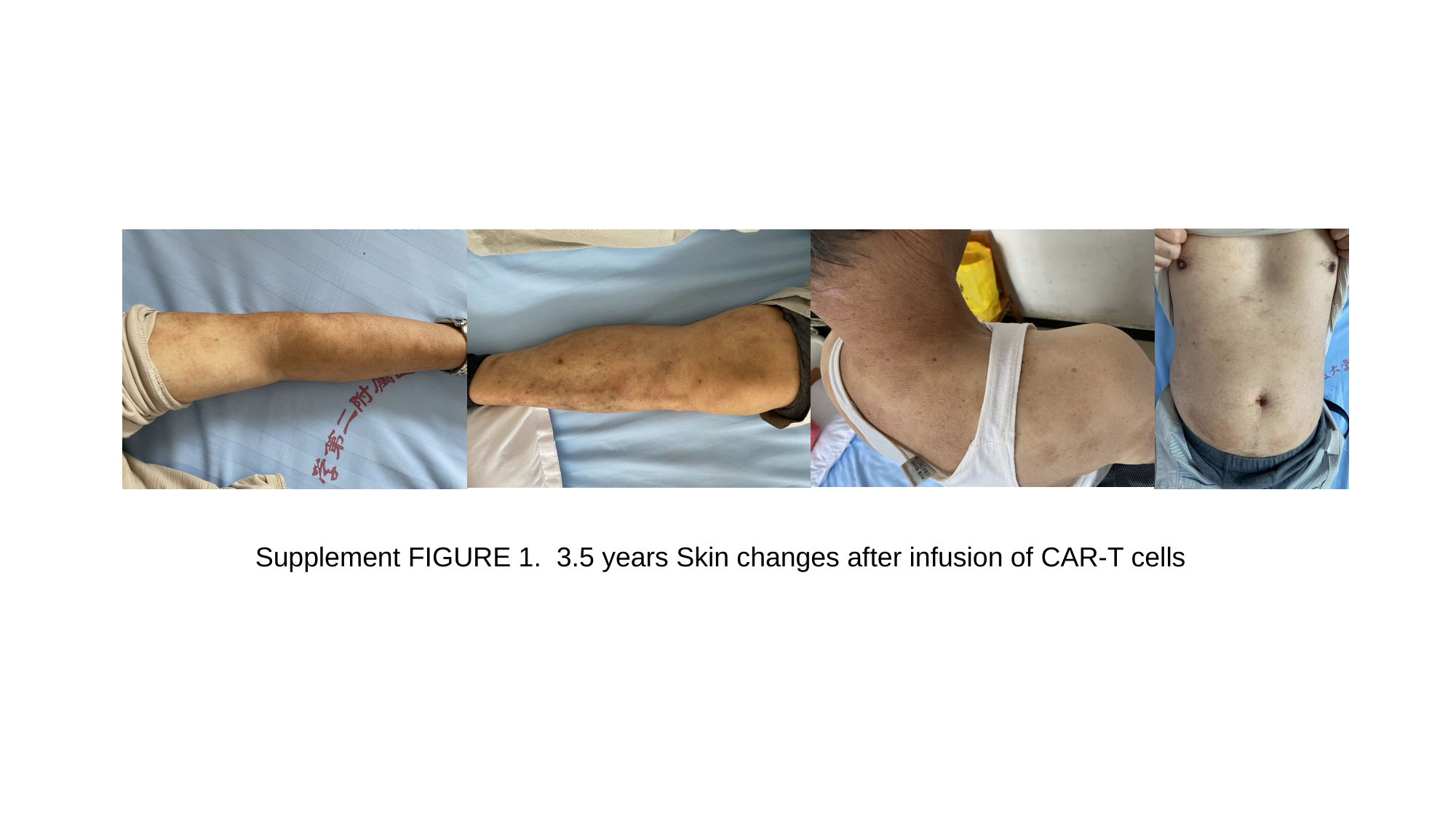

Supplement FIGURE 1. 3.5 years Skin changes after infusion of CAR-T cells

Supplement: Supplementary file 1 [file Presentation1.pptx]
